# Quantifying and modeling loss of estrogen and progesterone in PDMS-based devices

**DOI:** 10.1007/s10404-025-02852-1

**Published:** 2025-11-03

**Authors:** Nathaniel G. Hermann, Richard A. Ficek, Dmitry A. Markov, Lisa J. McCawley, M. Shane Hutson

**Affiliations:** 1 Department of Physics and Astronomy, Vanderbilt University, Nashville, 37240 TN USA; 2 Department of Biomedical Engineering, Vanderbilt University, Nashville, 37240 TN USA

**Keywords:** Polydimethylsiloxane, Microfluidics, Steroid hormones, Estrogen, Progesterone, Organ-on-a-chip

## Abstract

An early study on the biological consequences of using polydimethysiloxane (PDMS) for microfluidic cell culture reported that estrogens could be sequestered by PDMS; however, the PDMS interaction parameters of specific hormones have not been reported. Without these parameters, it is not possible to assess whether such sequestration is a problem for a particular device and flow rate combination. Here we quantify chemical-PDMS interactions for a commonly used estrogen and two additional steroid-class hormones: estradiol, aldosterone, and progesterone. We find that aldosterone does not detectably interact with PDMS; estradiol interacts modestly; and progesterone interacts strongly. Based on these measured interactions, we computationally model dynamic dosing protocols based on pulsed/bolus delivery and circadian control. We show that interactions with PDMS can strongly disrupt these dynamic dosing protocols in a chemical-specific and flow-rate-dependent manner. Notably, estradiol-PDMS interactions can have significant impacts under static conditions or low flow rates, but those impacts become negligible at higher flow rates. These results have critical implications for the use of steroid hormones in PDMS-based microfluidic devices.

## Introduction

Many miniaturized biomedical devices, including lab-on-a-chip, organ-on-a-chip (OOC), and microphysiological systems (MPS) are fabricated from polydimethylsiloxane (PDMS); however, PDMS is known to interact with hydrophobic chemicals (McDonald et al. 2000; Toepke and Beebe 2006; Wang et al. 2012; Meer et al. 2017; Regehr et al. 2009; Auner et al. 2019; Kemas et al. 2024; Hermann et al. 2025). Users of such devices need to know whether PDMS is significantly depleting hydrophobic components of the cell culture media. For example, early studies showed that cells cultured in certain PDMS-based devices were insensitive to added estrogen because it was sequestered by PDMS and no longer bioavailable (Regehr et al. 2009). These results caused great concern among users of PDMS-based devices, but they were not quantified in a way that enabled extrapolation to other device geometries, media-to-PDMS ratios, and media flow rates. Here, we investigate and quantify chemical-PDMS interactions for the estrogen estradiol and two other steroid-based hormones, aldosterone and progesterone. Importantly, these interactions are parameterized in terms of partition and diffusion constants that can be used in finite-element models for any user-defined device geometry under either static culture conditions or continuous perfusion.

The three steroid hormones investigated here are commonly used as growth factors in cell culture media (El-Ali et al. 2006; Young and Beebe 2010; Tehranirokh et al. 2013; Halldorsson et al. 2015; Leung et al. 2022). They are also a direct or indirect target of study for OOC/MPS-based research in reproductive biology, *e.g.* in endometrium-on-a-chip, fetal-membrane-on-a-chip, or mammary-gland-on-a-chip devices (Gnecco et al. 2017; Nawroth et al. 2018; Gnecco 2018; Mancini and Pensabene 2019; Park et al. 2021; Richardson et al. 2020; Ahn et al. 2021; Young and Huh 2021; Bodke and Burdette 2021; Sharma et al. 2022; Despicht et al. 2023; Moccia et al. 2023; Park et al. 2024; Vidal et al. 2024). Quantification of specific hormone-PDMS interactions is needed to properly assess in-device dosing, which is the first key step in any in-vitro-to-in-vivo extrapolation (Hermann et al. 2025).

The need to quantify and model hormone-PDMS interactions is further exacerbated by several use cases with dynamic hormone dosing. In the simplest cases, a pulse or step change in hormone levels may be used to trigger a change in cell behavior or differentiation (Chung et al. 2005; Matsumura et al. 2014; Yoshimitsu et al. 2014; Kashani et al. 2021; Miller et al. 2021), but reversible interactions with PDMS could yield reduced-amplitude pulses with long tails of low-level dosing. In more complicated scenarios, an experiment may require the modulation of hormone levels to match circadian rhythms (Cyr et al. 2017) or menstrual cycles (Gnecco et al. 2017; Xiao et al. 2017). In these periodic cases, interactions with PDMS can alter both the amplitude and phase of hormone dosing. Finally, for an experiment that measures the cellular secretion of a hormone over time, an appropriate model of hormone-PDMS interactions is needed to convert measured concentrations to dynamic secretion rates. There are strategies to mitigate hormone-PDMS interaction, involving either surface modification of PDMS (*e.g.* plasma oxidation) or inclusion of carriers in media. Surface modification is an imperfect solution: plasma oxidation is known to be transient, yielding PDMS surfaces with time-dependent hydrophobicity (Kim et al. 2004). This is particularly problematic for commercial devices, which may be stored before use for indeterminate times. On the other hand, the addition of carriers through methods such as using cell culture media with fetal bovine serum (FBS), can stabilize the concentration of hydrophobic growth factors (Fischer et al. 2019), but these strategies also alter dosing dynamics and require high fractions (2–10%) of FBS in media. They are also not amenable to applications that require serum-free media.

The three steroid hormones tested here are structurally similar, but their properties differ enough to expect them to interact with PDMS to different degrees. Aldosterone is a mineralocorticoid, estradiol is an estrogen, and progesterone is a progestogen, but their structures all share a gonane core. They also all share similar molecular weights (360.4, 272.4, and 314.5 amu) (Kim et al. 2023) and collisional cross sections (181.09, 173.34, and 182.2 Å^2^) (Zhou et al. 2016). The key difference is that aldosterone is much less hydrophobic than estradiol or progesterone, as demonstrated by their logP values of 1.08, 4.01, and 3.87 (Kim et al. 2023). Prior studies have linked lower values of logP with limited PDMS interaction (Auner et al. 2019; Hermann et al. 2025), and as expected, aldosterone is shown to not significantly interact with PDMS. Both estradiol and progesterone are shown to interact with PDMS, but contrary to their similar logP values, progesterone interacts much more strongly. This may be explainable through hydrogen bond donor count, which has been previously implicated in weakening PDMS interactions (Auner et al. 2019), given that estradiol has two hydrogen bond donors, while progesterone has none.

Taking a broader view of the state of biomedical microfluidic technology, problems of poor control of in-device dosages is an outstanding concern beyond just these hormones. Given the potential of these biomedical devices as new approach methods in medicine, and the lack of a robust regulatory framework for *in vitro* results, new approaches to estimating and validating in-device dosages are a crucial need. Here we extend previous methods to show that key interaction parameters can be determined by simple, static experiments and then applied to *in silico* simulations of complex, dynamic systems as a promising approach to validate in-device results.

## Materials and methods

### PDMS preparation

PDMS Sylgard 184 (Dow Corning, Auburn, MI) was mixed in a 10:1 mass ratio of elastomer base to curing agent. PDMS membranes were spun out from small volumes of PDMS to 80-$\upmu$m thickness on a Laurell WS-400-6NPP Spin Coater (Laurell Technologies Corporation, Lansdale, PA) using the procedure described by Markov et al. (Markov et al. 2014), and then cured overnight in a 67 $^{\circ }$-C oven.

### Hormone preparation

Aldosterone, estradiol, and progesterone were acquired as powders from Sigma Aldrich (St. Louis, MO). Stock solutions were prepared in neat dimethyl sulfoxide (DMSO) at approximately 70 mM concentration. Aqueous solutions were then prepared via a dilution of DMSO stock in 1x pH-7.4 phosphate buffered saline (PBS) (Thermo Fisher, Waltham, MA) to concentrations between 0.1 and 5 mM.

### Diffusion-through-membrane experiments

**Fig. 1:**
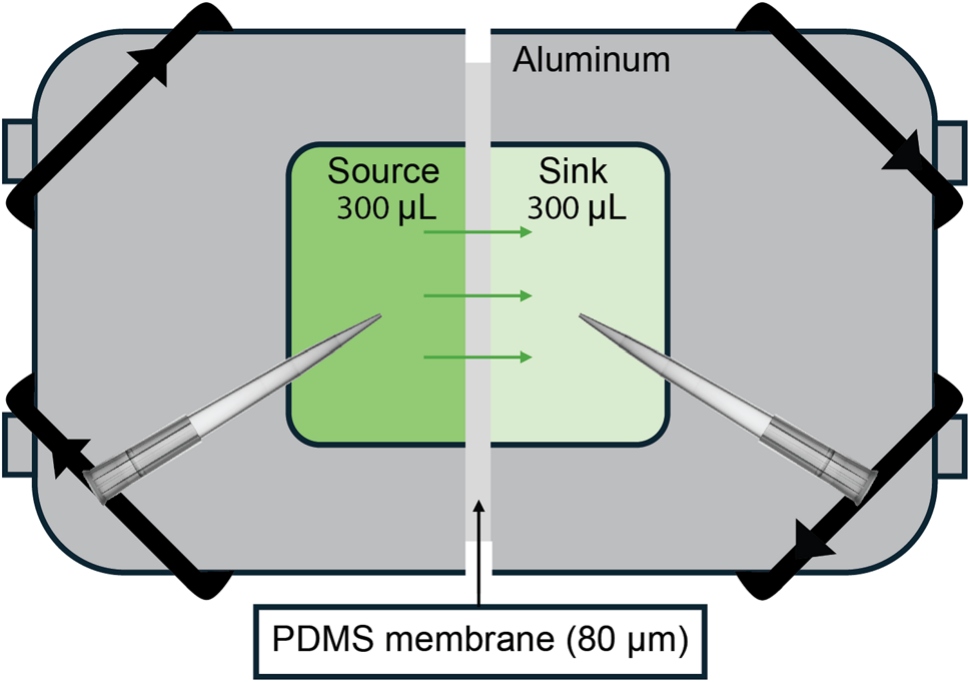
Schmetic of diffusion-through-membrane experiment. Aluminum chambers are filled with 300 µL of hormone solution (source) or blank solvent mixture (sink). Chambers are separated by an 80-µm thick PDMS membrane and sampled over 24 hours with UV-Vis spectroscopy performed on samples. Chambers are kept well-mixed on a blot mixer throughout

To determine chemical-PDMS interactions, we follow the procedure for diffusion-through-membrane experiments as described in (Hermann et al. 2025). In brief, two chambers separated by an 80-$\upmu$m thick PDMS membrane were each filled with 300 $\upmu$L of solution – one containing a hormone solution (source) and one containing a blank solvent mixture (sink) (Fig. 1). Each chamber was then sampled multiple times over 24 hours and UV-Vis spectroscopy was used to track hormone concentrations in both source and sink. Interaction parameters were estimated by fitting the dynamic source and sink concentrations to a mass-transport model, with numerical solutions to the model generated in COMSOL Multiphysics (COMSOL Inc., Burlington, MA) and fitting conducted in Mathematica (Wolfram Inc., Champaign, IL).

### Simulations of dynamic hormone exposures

To estimate how PDMS interactions would modulate dynamic hormone exposures, we used COMSOL Multiphysics. Using the finite element method (FEM), time-independent solutions for the velocity field were developed through the *laminar flow* module, and used in turn to develop time-dependent solutions for the concentration in solution and PDMS using the *transport of diluted species* module. Simulations were run both for static channels to emulate the results of Regehr et al. (2009) and for aqueous solutions of both progesterone and estradiol flowing through PDMS microchannels under pulse-chase and circadian rhythm scenarios.

## Results

**Fig. 2:**
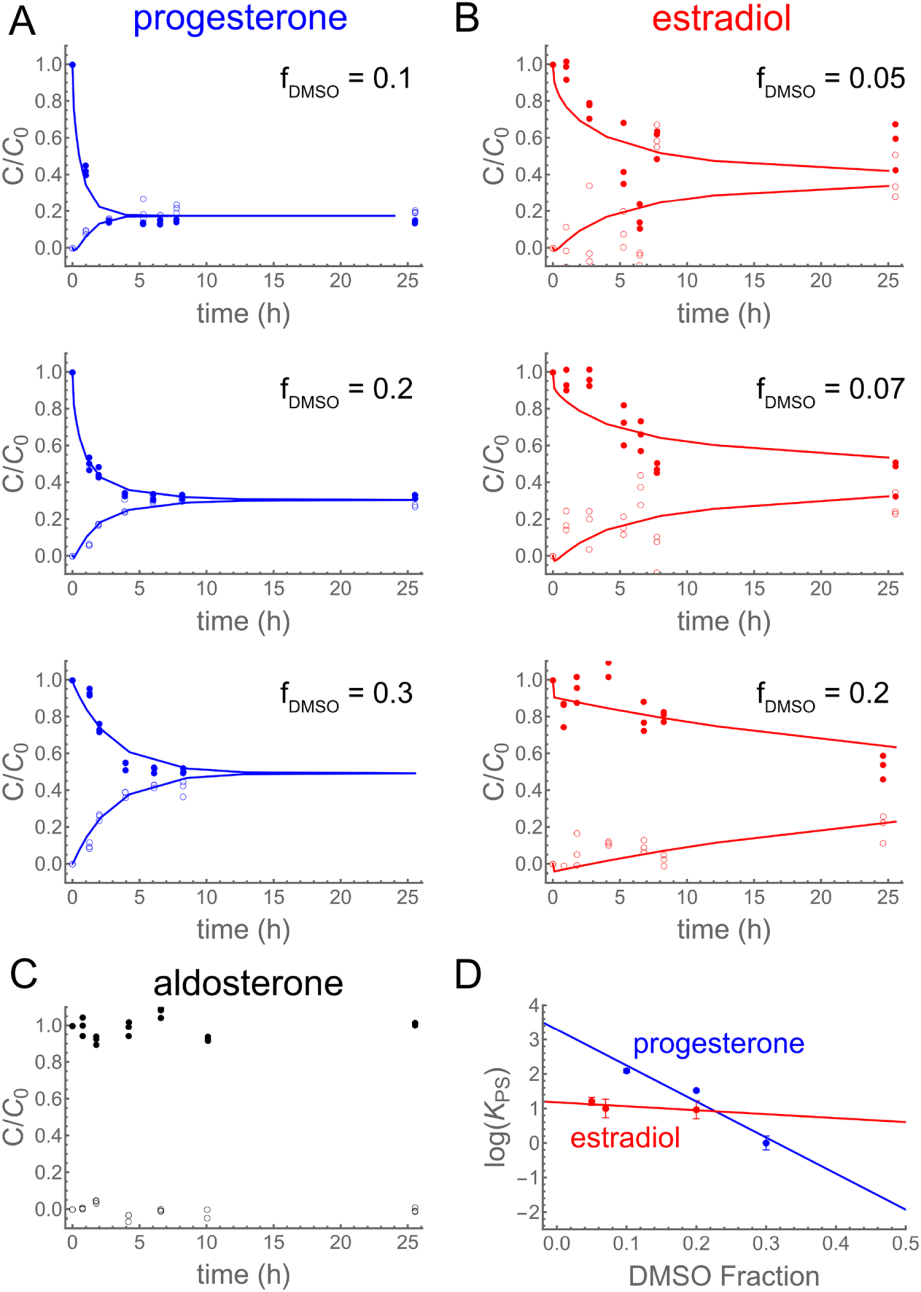
Hydrophobic steroid hormones partition into and diffuse through PMDS. (**A**–**C**) Diffusion-through-membrane results for (**A**-blue) progesterone, (**B**-red) estradiol, and (**C**-black) aldosterone. Filled symbols are concentrations in the source chamber; open symbols are concentrations in the sink chamber; lines are best fits to a partition-diffusion model. For progesterone and estradiol, the DMSO co-solvent fraction is as shown (increasing top to bottom). (**D**) Extrapolation of progesterone and estradiol results to zero DMSO based on a linear regression of fitted values of $\log {K_{PS}}$ versus DMSO fraction

As shown in Fig. 2A-B, both progesterone and estradiol partition into and diffuse across an 80-$\upmu$m thick PDMS membrane, with the source and sink chambers approaching equilibrium over hours to days. In contrast, aldosterone does not cross the membrane (Fig. 2C). To quantitatively characterize chemical-PDMS interactions for progesterone and estradiol, we fit this data to a partition-diffusion model (Hermann et al. 2025) to determine four key parameters: the effective diffusivity in solution ($D_{S,eff}$); diffusivity in PDMS ($D_{P}$); a mass-transfer coefficient at the solution-PDMS interface (*H*); and the PDMS-solution partition coefficient ($K_{PS}$). This model calculates concentration profiles in solution ($c_{S}$) and in PDMS ($c_{P}$), taking into account both advection in solution and diffusion in both solution and PDMS:

1 $$\begin{aligned} \frac{\partial c_{S}}{\partial t} = D_{S}\nabla ^{2}c_{S} -\nabla \cdot (c_{S}\textbf{v}) \end{aligned}$$

2 $$\begin{aligned} \frac{\partial c_{P}}{\partial t} = D_{P}\nabla ^{2}c_{P} \end{aligned}$$

and partitioning at the solution-PDMS interface ($\partial \Omega$) that determines boundary fluxes:

3 $$\begin{aligned} \hat{\textbf{n}}\cdot \nabla c_{S}(\textbf{r}\in \partial \Omega ) = -\frac{H}{D_{S}}(K_{PS}c_{S}-c_{P}) \end{aligned}$$

4 $$\begin{aligned} \hat{\textbf{n}}\cdot \nabla c_{P}(\textbf{r}\in \partial \Omega ) = +\frac{H}{D_{P}}(K_{PS}c_{S}-c_{P}) \end{aligned}$$

Here, $\textbf{v}$ is the velocity field in solution, and $D_{S}$ is the ideal diffusivity in solution. This model allows a user to simulate a device with any geometry and flow rate given known chemical-PDMS interaction parameters.

To get spectroscopically detectable concentrations of estradiol and progesterone into solution, we conducted these experiments with 5–30% DMSO as a cosolvent. We used a previously derived log-linear relationship (Hermann et al. 2025) to extrapolate from the partition coefficients in mixed solutions, $K_{PS}$, to estimate the partition coefficients in pure aqueous solution, $K_{PW}$. That relationship is

5 $$\begin{aligned} \log {K_{PS}} = f\log {K_{PD}} + (1-f)\log {K_{PW}}, \end{aligned}$$

where *f* is the volume fraction of DMSO and $K_{PD}$ is the PDMS-DMSO partition coefficient. The log-linear fits to the data are shown in Fig. 2D. The best-fit values of $K_{PW}$, $K_{PD}$, and $D_{P}$, plus a non-rate-limiting lower bound for *H*, are shown in Table 1. We find that progesterone partitions into PDMS two orders of magnitude more strongly than estradiol, and diffuses within PDMS about one order of magnitude faster.

Note that these experiments were conducted on a blot mixer to keep solutions well mixed. The fits thus estimate effective diffusion coefficients in solution, $D_{S,eff}$, which are not generally applicable for further device simulations. We instead report calculated values of $D_{S}$ based on the methods of Miyamono and Shimono (Miyamoto and Shimono 2020) with van der Waals radii estimated using the molecular descriptor package Mordred (Zhao et al. 2003; Moriwaki et al. 2018). These values of $D_{S}$ (1.98 - 2.17 mm^2^/h) are about an order of magnitude smaller than the self-diffusivity of water (10.8 mm^2^/h) (Tsimpanogiannis et al. 2019) and are reported as $\log {D_{S}}$ values in Table 1.

**Table 1:** Chemical-PDMS interaction parameters for steroid hormones

Chemical	$\log {K_{PW}}$	$\log {K_{PD}}$	$\log {D_{P}}$ (mm^2^/h)	$\log {D_{S}}$ (mm^2^/h)	$\log {H}$ (mm/h)
Progesterone	$3.30\pm 0.60$	$-7.20\pm 0.60$	$-1.90\pm 0.40$	0.336	$\ge 1.56$
Estradiol	$1.18\pm 0.14$	$+0.03\pm 0.14$	$-3.03\pm 0.32$	0.306	$\ge 1.56$
Aldosterone	–	–	–	0.296	–

### Simulations

To investigate and illustrate the impact of steroid-PDMS interactions, we used finite-element modeling to simulate the distributions of progesterone and estradiol in three microfluidic use cases. First, to demonstrate the agreement of our model with the prior results of Regehr et al. (2009), we simulated the system described therein: a 1 nM solution of estradiol was loaded into a 4.5-mm long, 670 µm-wide, and 250 µm-tall microchannel embedded in a 750 µm-tall, 10-mm wide, and 10-mm long slab of PDMS and held under static conditions for 24 hours; the channel was then emptied, reloaded with fresh solution and allowed to sit for an additional 24 hours. Our COMSOL simulations predict that 98.6% of the estradiol would be lost from solution after the first 24 hours, and that a freshly loaded solution would lose a similar amount over the next 24 hours. Both predictions match the experimental results to within 10%: Regehr et al. found that more than 90% of the initial estradiol was lost from solution after each 24 hour period. Thus, results from the model echo those of the original experiments: PDMS surfaces are not easily saturated; and loss of estradiol from solution into PDMS can be severe under static conditions.

**Fig. 3:**
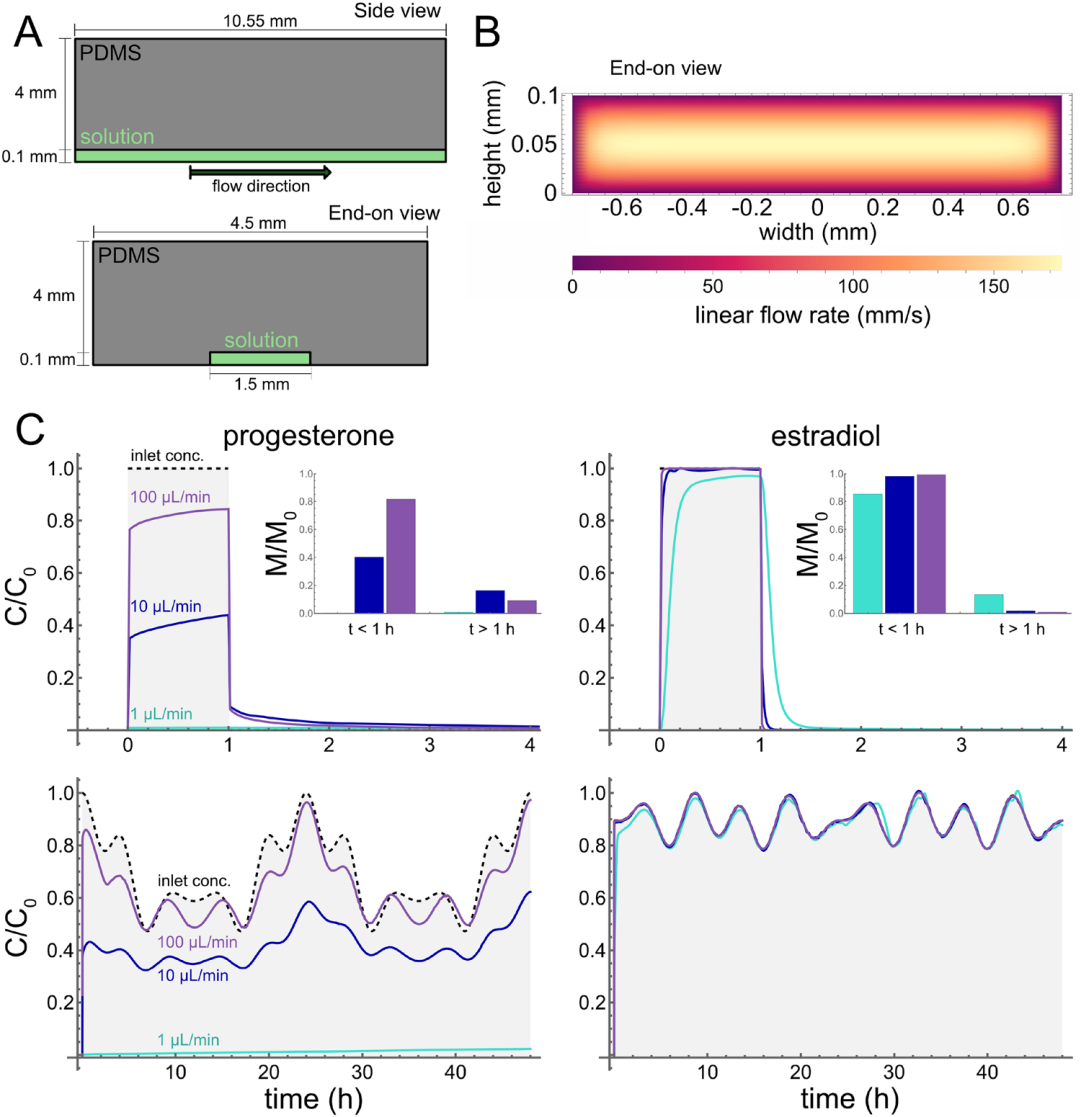
(**A**) Schematic of simulated microchannels as viewed from the top side or end-on. (**B**) Simulated linear flow velocity in the microchannels at volumetric flow rate $Q = 100$ µL/min. Flow is taken to be out of the page. (**C**) The fidelity of dynamic dosing protocols is disrupted by chemical-PDMS interactions in a flow-rate-dependent manner: (left column) progesterone; (right column) estradiol; (top row) simulations of a 1-hour pulsed dose; and (bottom row) simulations of circadian variations in hormone levels. Shaded regions with dashed outlines correspond to inlet concentrations. Colored lines correspond to average concentration in channel for volumetric flow rates of 1, 10 and 100 µL/min. Inset plots show normalized, integrated total amount of hormone delivered during the pulse ($t<1$ h) and following the pulse ($t>1$ h)

However, adding flow can change the situation dramatically. To illustrate the impact of flow, we simulated 1-hour pulses of each hormone as delivered through a rectangular microchannel: 10.55-mm long by 1.5-mm wide by 100-µm tall and embedded in a 4-mm thick slab of PDMS (Fig. 3A). These channels are a reasonable geometry for general microfluidics use and the exact geometry of the channels is taken from a thick-tissue bioreactor developed as a PDMS-based organ-on-chip device (Markov et al. 2012). These simulations were performed with volumetric flow rates, *Q*, of 1, 10 and 100 µL/min. Given the geometry of the channels, flow was assumed to be laminar, Poiseuille flow (Fig. 3B). These flow rates are chosen to be within the wide range of flow rates (0.001 to 1000 µL/min) used in OOC devices (Cavaniol et al. 2022). As shown in Fig. 3C (top), the slowest of these flow rates yielded the strongest departures from the input square pulse. For strongly-interacting progesterone at that flow rate, less than 0.1% of the chemical reaches the end of the microchannel. For moderately-interacting estradiol, almost all the chemical reaches the end of the microchannel, but its interactions with PDMS round off the square pulse and lead to an extended delivery of hormone for about half an hour after the nominal end of the pulse. At faster flow rates, the delivered pulses of estradiol are only slightly rounded off, but those for progesterone remain limited to about 40% ($Q = 10$ µL/min) or about 80% ($Q = 100$ µL/min) of the input dose. Progesterone also has a low-dose, post-pulse delivery that lasts for a few hours.

For the final use case, we simulated delivery through the same microchannel but with an input concentration that follows a prescribed circadian rhythm. The different rhythms chosen for progesterone and estradiol are Fourier-smoothed versions of data from an *in vivo* study (Bungum et al. 2013). As shown in Fig. 3C (bottom), moderately-interacting estradiol can be delivered similarly to the targeted circadian rhythm at all tested flow rates. In contrast, strongly-interacting progesterone can be delivered somewhat closely to the targeted rhythm only at the highest flow rate (100 µL/min). Decreasing the flow rate to 10 µL/min reduces the circadian variation by about half; decreasing to 1 µL/min completely eliminates the ability to follow the prescribed dynamics.

## Discussion

As we have shown, both estradiol and progesterone interact with PDMS, while aldosterone does not. The other two steroids have logP well above this threshold and do interact with PDMS, albeit in distinct ways. Progesterone strongly partitions into PDMS ($K_{PW}\sim 1990$) and diffuses through bulk PDMS at a moderate rate ($D_{P}\sim 0.013$ mm^2^/h). Estradiol partitions more weakly ($K_{PW}\sim 15.2$) and diffuses through PDMS more slowly ($D_{P}\sim 9.33\times 10^{-4}$ mm^2^/h). These differences are notable given the hormones’ similar logP and molar mass.

Under static culture conditions, even estradiol’s modest partitioning into PDMS can lead to its severe depletion from the media, as observed by Regehr et al. (2009); however, simulations of pulsed or bolus delivery of these steroid hormones show that depletion is strongly impacted by flow rate. We may additionally consider the total amount of hormone reaching the channel outlet *M*, calculated from averaged outlet concentration

6 $$\begin{aligned} M(t) = \int _{0}^{t}Qc(\tau )d\tau \end{aligned}$$

Given our simulated pulse length of 1 h, we find that total progesterone dosing during the pulse is strongly depleted even at high flow rates (18.4% depletion at $Q=100$ µL/min, 60.7% depletion at $Q=10$ µL/min) with extended release following the pulse (9.1% released at $Q=100$ µL/min, 16.3% released at $Q = 10$ µL/min). Total estradiol dosing during the pulse is only weakly disrupted at low flow rates (14.7% depletion at $Q=1$ µL/min, 1.9% depletion at $Q=10$ µL/min) with similarly moderate extended release (13.5% released at $Q=1$ µL/min, 1.8% released at $Q=10$ µL/min) (Fig. 3C). Considering instantaneous concentration at the channel outlet, at low flow rates ($Q = 1$ µL/min), progesterone is strongly depleted from solution (only 0.03% is bioavailable at the end of the 10.55-mm channel), and a pulse of estradiol is rounded off and extended by a slow release of hormone from the microchannel walls well after the end of the pulse (0.1-10% of the initial dosage is bioavailable at the end of the 10.55-mm channel during the first 3 hours following the pulse). At higher flow rates, the system shifts into an advection-dominated regime, resulting in much more progesterone and nearly all estradiol reaching the target. Using higher flow rates can thus mitigate the effects of chemical-PDMS interactions, but only if cells in the device can tolerate the higher flow rates.

Simulations of circadian hormone delivery with progesterone and estradiol suggest that in-device dosing requires careful management to account for progesterone-PDMS interaction. At low flow rates ($Q = 1$ µL/min), interactions with PDMS severely limit in-device dosing of progesterone (less than 2.4% of targeted exposure after 48 hours) and dampen any periodic behavior. At faster flow rates ($Q = 10-100$ µL/min), more than 30% of the progesterone is delivered through the channel, but its periodic behavior is phase-shifted. Further, in the case of 10 µL/min flow rates, the amount of progesterone delivered increases with each 24-hour cycle. On the other hand, weaker interactions with PDMS allow allow nearly complete delivery of estradiol in a circadian rhythm at all tested flow rates. These simulations raise concerns about the feasibility of circadian (or menstrual) control of hormone concentrations in PDMS-based devices with naive aqueous solution or serum-free media. For strongly-interacting chemicals and physiologically-relevant flow rates, it seems likely that the addition of serum, carriers, or co-solvents will be necessary to avoid severe hormone depletion or phase-shifting.

Taking a broader view of the state of research in PDMS-based devices, we note that the steroid hormones discussed here are a tiny subset of the cell culture components that could interact strongly with PDMS. Typical culture media include a range of hydrophobic hormones, vitamins, lipids, and growth factors. These are often introduced as components of added serum, and while the carrier proteins in serum do help mitigate loss of these hydrophobic compounds into PDMS (Fischer et al. 2019), the composition of the serum is often poorly defined. For PDMS-based devices, it may be advisable to use chemically-defined, serum-free media. With a well-defined medium and measured chemical-PDMS interaction parameters, one can use 3D chemical transport modeling to ensure that the in-device concentrations of relevant compounds are known and controlled. The methods used here are one example of a general approach needed across many microfluidic applications – *i.e.* deriving parameters from simple, static experiments and using these to construct dynamic simulations – that are appropriate for the design design and validation of microfluidics assays.

## Conclusions

We have determined that the steroid hormones estradiol and progesterone both partition into and diffuse through PDMS; a third less hydrophobic steroid, aldosterone, does not. By parameterizing the interactions of estradiol and progesterone with PDMS, we have developed simulations of pulsed or bolus delivery and circadian concentration control in PDMS-based devices: Both dosing schemes can be compromised by hormone-PDMS interactions. Impacts on estradiol delivery are only significant at flow rates below µL/min. In contrast, impacts on progesterone are severe at low flow rates and remain significant even up to 100 µL/min. Researchers interested in applying dynamic dosing of these hormones in PDMS-based microfluidic systems can use multiphysics FEM simulations to account for and plan strategies to mitigate in-device hormone loss.

## Data Availability

Data for this article, including the results of membrane experiments and simulated solutions used in generating interpolation functions, are available at Open Science Framework at https://osf.io/z5bvx/.
